# Olanzapine-Induced Weight Gain and Glycolipid Metabolism Aberrations in First-Episode and Antipsychotic-Naïve Schizophrenia Patients: A Longitudinal Study

**DOI:** 10.2174/1570159X23666240918103730

**Published:** 2024-09-20

**Authors:** Shen Li, Doudou Zheng, Wanyao Wang, Nannan Liu, Yanzhe Li, Chenghao Lu, Yeqing Dong, Xinxu Wang, Wei-Dong Li, Jie Li

**Affiliations:** 1 Institute of Mental Health, Tianjin Anding Hospital, Mental Health Center of Tianjin Medical University, Tianjin, 300222, China;; 2 Psychoneuromodulation Center, Tianjin Anding Hospital, Mental Health Center of Tianjin Medical University, Tianjin, 300222, China;; 3 Department of Genetics, College of Basic Medical Sciences, Tianjin Medical University, Tianjin 300070, China

**Keywords:** Schizophrenia, olanzapine, weight gain, glycolipid metabolism, first-episode, antipsychotic-naïve, insulin

## Abstract

**Objective:**

Limited research has delved into the comprehensive impact of monotherapy on weight and glycolipid metabolism in schizophrenia (SCZ) patients. Our study aims to longitudinally investigate the multidimensional effects of olanzapine (OLA) monotherapy on weight and glycolipid metabolism in first-episode and antipsychotic-naïve (FEAN) SCZ patients.

**Methods:**

A total of 74 FEAN-SCZ patients were recruited, as well as 58 sex- and age-matched healthy controls. Eligible patients underwent a 4-week OLA treatment regimen, with weight assessments conducted at baseline and week 4. Moreover, lipid profiles and fasting plasma glucose (FPG) were measured at baseline and week 4. Insulin, leptin (LEP), and adiponectin (APN) levels were determined using enzyme-linked immunosorbent assay (ELISA) kits.

**Results:**

At baseline, FEAN-SCZ patients showed elevated levels of insulin, low-density lipoprotein (LDL), impaired insulin sensitivity, and reduced levels of APN compared to the healthy controls. Following 4-week OLA treatment, patients showed an increase in body mass index (BMI) of 0.96 kg/m^2^. Additionally, FPG, quantitative insulin sensitivity check index (QUICKI), HOMA-insulin sensitivity index (HOMA-ISI), and fasting plasma glucose to insulin ratio (G/I) displayed significant decreases, while insulin, HOMA-IR, and LEP levels showed significant increases. Stepwise regression analysis revealed that baseline FPG independently predicted the change in BMI after 4 weeks of OLA treatment.

**Conclusion:**

FEAN-SCZ patients exhibited pre-existing alterations in glucose homeostasis. After 4 weeks of OLA treatment, SCZ patients experienced significant weight gain, deteriorating insulin resistance, and increased LEP levels. In addition, baseline FPG emerged as a predictor of BMI changes after 4 weeks of OLA treatment.

## 1 INTRODUCTION

The metabolic challenges faced by patients with schizophrenia (SCZ) impose significant burdens on themselves, their families, and society at large [1]. These metabolic disturbances are frequently associated with the use of antipsychotic medications [2]. Initially, the introduction of antipsychotics represented a breakthrough in managing SCZ symptoms and reducing recurrent hospitalizations [3]. Currently, the first-line antipsychotics in clinical practice are second-generation antipsychotics, including olanzapine (OLA), clozapine (CLO), and risperidone, collectively known as atypical antipsychotic drugs (AAPD), which exhibit excellent efficacy. Unfortunately, some AAPDs are also associated with metabolic side effects, such as weight gain, hyperglycemia, dyslipidemia, and cardiovascular complications [4]. OLA and CLO are known to induce substantial changes in body weight and glucolipid metabolism [5]. Thus, the impact of AAPD on promoting weight gain and metabolic irregularities in SCZ patients should not be underestimated [6]. These metabolic side effects due to AAPD have emerged as a leading cause of premature mortality among SCZ patients [7]. As such, there is an urgent imperative to unravel the underlying mechanisms through which AAPD contributes to weight gain and disruptions of glucolipid metabolism.

Previous studies have yielded contradictory results regarding the impact of AAPD on fasting plasma glucose (FPG). Some studies have concluded that FEAN-SCZ patients treated with AAPD intervention exhibited no significant change in FPG from pre-treatment levels [8-10]. However, one study [11] observed a significant increase in FPG following AAPD treatment. Some studies failed to identify differences in relevant glycolipid metabolism indicators between SCZ patients and healthy population at baseline [12, 13], while another study [14] did not find significant differences in triglycerides (TG), high-density lipoprotein (HDL) and low-density lipoprotein (LDL) between FEAN-SCZ patients and healthy controls. Nevertheless, some researchers have reported inconsistent findings, *i.e*., significant increases in LDL and TG, along with significant decreases in HDL in SCZ patients after AAPD treatment [15, 16], and consistent results have been obtained from animal studies [17].

Recent studies indicate that AAPD may directly influence adipose tissue, thereby disrupting body energy homeostasis [18]. Adipose tissue dysfunction can lead to abnormal production and secretion of adipokines, which are known contributors to obesity and metabolic syndrome development [19]. Adipokines, including adiponectin (APN) and leptin (LEP), play pivotal roles in weight gain and metabolic disorders induced by AAPD [20, 21]. A Meta-analysis suggests that APN levels in SCZ patients do not significantly differ from those in healthy controls; however, plasma APN levels significantly decrease with AAPD treatment [22]. The precise mechanism underlying the impact of AAPD on APNs remains unclear, though some studies suggest that APN is related to the regulation of glucolipid metabolism [23]. Previous research by our group has indicated that APN gene polymorphisms are associated with AAPD-induced increases in body weight and waist-hip ratio [24].

Our own findings revealed that SCZ patients with glucose homeostasis disturbances even before the initiation of treatment [25]. Impaired glucose tolerance may manifest independently of antipsychotic treatment [26]. It has been shown that first-episode and antipsychotic-naïve (FEAN) SCZ patients often exhibit an increased incidence of impaired glucose tolerance and heightened insulin resistance (IR) compared to healthy controls [27-29].

To our knowledge, no prior studies have systematically assessed alterations in glucolipid metabolism following OLA treatment in a comprehensive and multidimensional manner. Thus, the primary objective of this study was to provide an extensive understanding of the changes in weight gain and glucolipid metabolism induced by OLA treatment. The study focused on FEAN-SCZ patients as study subjects, with the aim of testing the following hypotheses: 1) FEAN-SCZ patients manifest changes in glucose homeostasis compared to matched healthy controls; 2) after a 4-week OLA treatment period, SCZ patients undergo weight gain and exacerbated abnormalities of glucolipid metabolism; 3) baseline glycolipid metabolic indicators may serve as predictors for changes in BMI after 4 weeks of OLA treatment.

## 2 MATERIALS AND METHODS

### 2.1 Participants

For the study, we enrolled 74 FEAN-SCZ patients (male/female: 27/47), aged between 18 and 45 years (average age: 25.45 ± 4.19 years) from Tianjin Anding Hospital. The average duration of disease in FEAN-SCZ patients was 17.70 ± 17.67 months. The inclusion criteria were as follows: 1) meeting the diagnosis of SCZ based on the Structured Clinical Interview for Diagnostic and Statistical Manual of Mental Disorders, Fourth Revised Edition (DSM-Ⅳ-TR) criteria (SCID); 2) experiencing the first episode with antipsychotic intervention lasting no more than 2 weeks in a lifetime; 3) duration less than 2 years; 4) current psychiatric symptoms rated above 4 on the Clinical Global Impression (CGI-S); 5) Han Chinese; 6) having an intelligence quotient (IQ) > 70. The exclusion criteria included: 1) organic brain injury, brain diseases, or other neurological diseases; 2) severe physical diseases. In conducting a controlled clinical trial, we recruited 58 sex- and age-matched healthy controls (male/female: 27/31; average age: 26.09 ± 2.62 years) from the local community. Inclusion criteria for controls stipulated the absence of a personal or family history of psychotic disorders, substance abuse, or previous chronic dependence.

All FEAN-SCZ patients underwent screening and evaluation by two independent, experienced psychiatrists. Prior to assessment, all participants provided written informed consent.

### 2.2 OLA Intervention

Eligible FEAN-SCZ patients were assigned to undergo a 4-week observation with OLA treatment. The OLA dosage commenced at 5 mg/day and was gradually adjusted to a range of 15-20 mg/day. Psychiatrists tailored the drug titration schedule based on clinical requirements. Throughout the study period, trihexyphenidyl was permitted solely for the management of extrapyramidal symptoms, and benzodiazepines were administered as needed for the treatment of insomnia. The diet, exercise, and lifestyle of all patients were managed by the hospital.

### 2.3 Demographic Characteristics

A self-designed questionnaire was utilized to collect general demographic data, including age, age of onset, education levels, sex, height, body weight, and other relevant factors. Body mass index (BMI), an index of weight-for-height widely used to classify overweight and obesity in adults, was calculated as an individual's weight in kilograms divided by the square of their height in meters (kg/m^2^).

### 2.4 Positive and Negative Syndrome

The severity of psychotic symptoms was assessed using the Positive and Negative Syndrome (PANSS) [30]. Two independent and experienced psychiatrists who underwent a pre-study training program evaluated the scale. The inter-rater correlation coefficient for repeated PANSS total score assessments exceeded 0.8 The scale comprises 30 items rated along a 7-point Likert scale (“1-7”), indicating absent, minimal, mild, moderate, moderate-severe, severe, and extreme, respectively. The PANSS comprises three subscales: positive symptoms, negative symptoms, and general psychopathological symptoms. The Chinese version of PANSS has demonstrated good reliability and validity [31]. The total PANSS score was computed based on all 30 items, with higher scores indicating more severe psychotic symptoms.

### 2.5 Metabolic Indexes Testing

Fasting blood samples were collected from the participants in the morning. Peripheral venous blood was drawn into an EDTA tube at baseline and at the 4-week follow-up. Using conventional direct methods and an automatic biochemistry analyzer (Olympus AU2700), FPG, total cholesterol (TCHO), TG, HDL, and LDL were measured. Biochemical indexes, including insulin, LEP, and APN, were detected using certified enzyme-linked immunosorbent assay (ELISA) kits (Elabscience Biotechnology, Co.).

### 2.6 Calculation of Indexes Related to Insulin Sensitivity Evaluation

Insulin sensitivity evaluation involves the calculation of various indexes, namely, quantitative insulin sensitivity check index (QUICKI), homeostatic model assessment for insulin resistance (HOMA-IR), HOMA-insulin sensitivity index (HOMA-ISI), and fasting plasma glucose to insulin ratio (G/I). These were computed as follows: QUICKI = 1/[log (FPG) + log (insulin)], HOMA-IR = (insulin×FPG)/22.5, HOMA-ISI = ln[1/(insulin×FPG)], and G/I = FPG/insulin.

### 2.7 Statistical Analysis

STATA 15 (StataCorp, College Station, Texas) and GraphPad Prism 9 (GraphPad Software Inc, La Jolla, USA) were used for statistical analyses. Categorical variables were presented as frequencies, while continuous variables were expressed in terms of mean ± standard deviation after the Kolmogorov-Smirnov test. Chi-square tests and analysis of variance (ANOVA) were employed for categorical and continuous variables, respectively, to assess differences in demographic characteristics and clinical variables between FEAN-SCZ patients and healthy controls. Multivariate analysis of covariance (MANCOVA), adjusted by sex, age, and BMI, was conducted to examine the differences in each variable between controls and patients. Multiple test correction was performed using a Bonferroni post hoc test. Stepwise regression analyses, with sex, age, and baseline BMI as covariates, were performed to predict changes in BMI from baseline variables. A significance level of *P* < 0.05 was considered statistically significant.

## 3 RESULTS

### 3.1 Demographic and Clinical Variables

To explore both the commonalities and differences in the pre-treatment metabolic characteristics between FEAN-SCZ patients and healthy controls, as well as to assess the similarities and differences in the metabolic characteristics of SCZ patients before and after 4 weeks of OLA treatment, relevant data was collected from 58 healthy controls and 74 FEAN-SCZ patients for analysis.

**Fig. (1):**
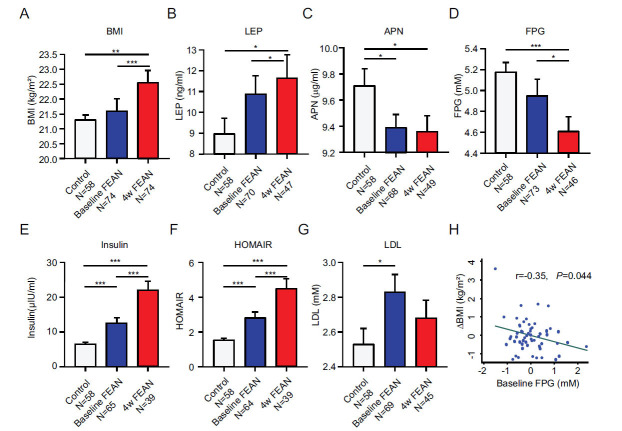
Glycolipid metabolism under OLA administration in FEAN-SCZ patients. (**A**) Average BMI for control (white, N=58), FEAN at baseline (blue, N=74) and FEAN at the 4-week follow-up (red, N=74). (**B**) Leptin levels (LEP) (white, N=58; blue, N=70; red, N=47). (**C**) Adiponectin (APN) (white, N=58; blue, N=68; red, N=49). (**D**) Fasting plasma glucose (FPG) (white, N=58; blue, N=73; red, N=46). (**E**) Insulin (white, N=58; blue, N=65; red, N=39). (**F**) Homeostasis model assessment insulin resistance index HOMAIR (white, N=58; blue, N=64; red, N=39). (**G**) Low-density lipoprotein (LDL) (white, N=58; blue, N=69; red, N=45). (**H**) Correlation between baseline FPG and the changes in BMI in FEAN-SCZ patients (N=73). (*P < 0.05; **P < 0.01; ***P < 0.001).

Analysis of demographic characteristics obtained from both groups revealed no significant differences in sex, age, education levels, and BMI in FEAN-SCZ patients compared with those in healthy controls (all *P*_s_ > 0.05). Furthermore, after a 4-week of OLA treatment, BMI increased significantly (*F*=-8.46, *P* < 0.001) (Table **1**, Fig. **1A**), psychotic symptoms improved, as evidenced by a significant decrease in the PANSS total scores (*F*=561.57, *P* < 0.001) (Table **1**) and the PANSS general psychopathological symptom scores (*F*=46.82, *P* < 0.001) (Table **1**, Fig. **1**).

### 3.2 Glucose Metabolism under OLA Administration in FEAN-SCZ Patients

In this study, the analysis of baseline data collected from both groups revealed significant differences in insulin, QUICKI, HOMA-IR, HOMA-ISI, and G/I in FEAN-SCZ patients compared with those in healthy controls (all *P_s_* < 0.001) (Table **1**, Figs. **1E** and **F**). However, no significant difference in FPG was observed between FEAN-SCZ patients and healthy controls (*P* > 0.05).

Upon comparing and analyzing metabolic and insulin sensitivity-related indices before and after 4 weeks of OLA treatment in FEAN-SCZ patients, we found that FPG showed a significant decrease (*F*=2.08, *P* = 0.043) (Table **1**, Fig. **1D**). Conversely, insulin and related indices demonstrated deteriorations, including QUICKI, HOMA-IR, HOMA-ISI, and G/I at the end of 4-week of OLA treatment period (all *P_s_* < 0.001) (Table **1**, Figs. **1E** and **F**).

### 3.3 Lipid Metabolism under OLA Administration in FEAN-SCZ Patients

At baseline, significant differences were observed in LDL and APN levels between FEAN-SCZ patients and healthy controls (Table **1**, Figs. **1C**, **1G**). Simultaneously, other lipid metabolic variables (TCHO, TG, HDL, and LEP) displayed no significant differences between FEAN-SCZ patients and healthy controls (all *P*_s_ > 0.05).

By comparing and analyzing lipid metabolic characteristics before and after 4 weeks of OLA treatment in FEAN-SCZ patients, it was observed that plasma LEP levels significantly increased after 4 weeks of OLA administration (*F*=-2.16, *P* = 0.036) (Table **1**, Fig. **1B**). Otherwise, other lipid metabolic variables (TCHO, TG, HDL, LDL, and APN) did not show significant changes after 4 weeks of OLA administration (all *P*_s_ > 0.05).

### 3.4 Prediction of Weight Gain under 4-week OLA Administration in FEAN-SCZ Patients

Regression models were constructed utilizing all glycolipid metabolic variables at baseline as independent variables, the change in BMI of SCZ patients before and after OLA treatment as dependent variables, and sex, age, and baseline BMI as covariates. Through stepwise regression analysis, absolute baseline FPG emerged as an independent predictor of the change in BMI after 4 weeks of OLA treatment (*β*=-0.24, *t*=-2.02, 95% CI=-0.47, -0.002, *P* = 0.048) (Fig. **1H**).

## 4 DISCUSSION

Despite the widespread use of OLA, there remains a dearth of studies systematically assessing the comprehensive and multidimensional alterations in glucolipid metabolism following its treatment. Thus, our study was to offer an extensive understanding of the intricate changes induced by OLA treatment, focusing on both weight gain and glucolipid metabolism. Key findings from this study underscored that FEAN-SCZ patients exhibited disturbances in glucose homeostasis, characterized by markedly elevated LDL levels and reduced APN levels compared to healthy controls. Following a 4-week OLA treatment period, SCZ patients experienced substantial weight gain, exacerbated IR, and heightened LEP levels. Notably, baseline FPG emerged as a predictive factor for changes in BMI after 4 weeks of OLA treatment.

### 4.1 Glycolipid Metabolism in FEAN-SCZ Patients

Contrary to prevalent findings in the literature [12-14], our results found a significant increase in LDL levels in FEAN-SCZ patients compared to healthy controls. This discrepancy may be attributed to variations in inclusion criteria across studies, with our study employing a more stringent definition of “first-episode” patients. Additionally, differences in sample sizes and the demographic composition, particularly focusing on the Chinese Han population in our study as opposed to the predominantly European and American populations in previous studies, could contribute to the observed distinctions.

In the evaluation of insulin sensitivity indexes, although no significant differences were noted in FPG levels between the two groups, FEAN-SCZ patients exhibited significantly higher insulin levels and HOMA-IR, along with significantly lower QUICKI, HOMA-ISI and G/I compared to healthy controls. This suggests a reduction in insulin sensitivity and an elevation in IR levels among SCZ patients not receiving antipsychotic drugs. These findings align with prior research [32, 33], indicating the existence of IR in SCZ patients even before initiating treatment with AAPD. Furthermore, our study identified lower APN levels in FEAN-SCZ patients compared to healthy controls, a finding at odds with a previous study [34]. The possible reasons for this were firstly related to the different ways of detecting APN and, secondly, the existence of different subtypes of APN in the body. Therefore, the intricate relationship between APN and SCZ warrants further in-depth exploration.

### 4.2 Glucose Metabolism under OLA Administration

Contrary to the majority of previous findings [35, 36], our study observed a significant decrease in FPG levels in SCZ patients after a 4-week treatment with OLA. Several factors could contribute to these disparate findings. Firstly, inconsistencies in inclusion criteria across studies may account for variations in patient profiles. The diverse characteristics of individuals included in each study could impact the observed changes in FPG levels. Secondly, differences in the duration of treatment observation might contribute to the conflicting results. Prior studies have employed varying observation periods, ranging from 6 weeks to over a year, whereas our study specifically focused on a 4-week timeframe. The detection of FPG at different time points could potentially yield disparate outcomes. Additionally, variations in sample traits and sizes among studies may influence the observed effects.

Notably, the observed decrease in FPG after OLA treatment prompts consideration of potential associations with negative glycemic feedback and IR. Biological mechanisms involving insulin signaling pathway-related substances may play a crucial role in the interplay between SCZ pathophysiology and the impact of AAPD on glucose metabolism [37, 38]. The exacerbation of glucose metabolism disorders by antipsychotic drugs could result from increased insulin resistance and decreased sensitivity [39, 40]. One study indicated a significant increase in insulin resistance in FEAN patients compared to patients on continuous medication [41]. The intricate relationship between antipsychotic drugs, insulin signaling, and glucose metabolism necessitates further exploration.

### 4.3 Lipid Metabolism under OLA Administration

In our study, a noteworthy increase in LEP levels was observed after a 4-week OLA treatment, potentially indicative of LEP resistance development. LEP resistance denotes reduced sensitivity to leptin in the brain, manifesting as a diminished capacity of LEP to enhance metabolism, reduce appetite, and subsequently contribute to increased food intake, leading to conditions such as overweight, obesity, cardiovascular disease disorders, and other metabolic disorders [42]. No significant alterations were noted in other lipid metabolism indicators in our study. Potential explanations for these outcomes may be related to specific inclusion criteria, the baseline conditions of the enrolled patients (*e.g*., relatively young mean age), or the duration of observation. Future studies could benefit from expanding sample sizes and extending the duration of observation to provide a more comprehensive understanding of the dynamic changes in lipid metabolism indicators. Addressing these aspects would contribute to obtaining a more representative portrayal of the nuanced effects of antipsychotic drugs on lipid metabolism.

### 4.4 Prediction of Weight Gain under OLA Treatment

The anticipation of post-treatment weight changes through the examination of baseline indicators preceding antipsychotic interventions is a current focal point for researchers. It was found that baseline FPG serves as a predictor for the subsequent change in BMI after a 4-week OLA treatment. AAPDs have the capacity to trigger alterations in factors related to the liver, as evidenced by the conversion of excess exogenous glucose to TG within the liver. These TG are subsequently packaged into LDL and transported to adipose tissue for prolonged energy storage [43]. The ability of AAPDs to increase blood glucose levels and induce insulin resistance may disrupt TG metabolism, contributing to dyslipidemia [44]. Pertinent animal experiments highlight the pivotal role of hepatocytes in maintaining glucose homeostasis through the regulation of glycolysis, gluconeogenesis, glucose uptake, and glycogen synthesis [45]. The intricate mechanism warrants further in-depth study. It is well known that patients with schizophrenia often experience weight gain during antipsychotic treatment [46]. This weight gain may lead to a decrease in treatment adherence, as patients may be dissatisfied with changes in their body shape, which can affect the normal use of medication. In addition, weight gain may be associated with adverse reactions and side effects of medication [47], which may prompt patients to discontinue medication. Therefore, in the management of antipsychotic treatment for patients with schizophrenia, it is important to pay attention to and actively manage the issue of weight gain, in order to ensure that patients can maintain treatment adherence and minimize the adverse effects of medication.

### 4.5 Limitations

Several limitations were inherent in our present investigation. Firstly, the sample size is limited and consists only of samples from the Han Chinese sample, which may affect the generalizability of the results. Second, the relatively brief duration of the antipsychotic treatment observation in SCZ patients might have contributed to discrepancies in results compared to prior studies. Future endeavors will involve extending the treatment follow-up period to provide a more comprehensive understanding and continued monitoring of various indicators.

## CONCLUSION

In conclusion, FEAN-SCZ patients demonstrated altered glycolipid metabolism and reduced APN at baseline compared to healthy controls. OLA treatment induced weight gain and dysfunction in glucose and lipid metabolism after 4 weeks, elevating the risk of associated complications. Moreover, the predictive capability of baseline FPG for changes in BMI following 4 weeks of OLA treatment suggests its potential involvement in the mechanisms underlying OLA-induced weight gain in SCZ patients, laying the groundwork for further investigations.

## Figures and Tables

**Table 1:** Demographic, clinic, and glycolipid metabolism characteristics between FEAN-SCZ patients and healthy controls at baseline and follow up.

**Variables**	**Baseline**				**Follow up-4w**		**Statistic (*P*)^a^**	**Statistic (*P*)^b^**
	**Controls**	**N**	**FEAN Patients**	**N**	**FEAN Patients**	**N**		
Age (years)	26.09 ± 2.62	58	25.45 ± 4.19	74	-	-	1.04(0.311)	-
Onset age (years)	-	-	23.24 ± 8.55	74	-	-	-	-
Sex (M/F)	27/31	58	27/47	74	-	-	1.36(0.243)	-
Education (years)	10.72 ± 3.19	58	10.55 ± 3.47	74	-	-	-	-
BMI (kg/m^2^)	21.31 ± 1.19	58	21.60 ± 3.50	74	22.56 ± 3.44	74	0.38(0.541)	**-8.46(<0.001)**
PANSS	-	-	139.36 ± 10.99	74	130.57 ± 24.64	65	-	**561.57(<0.001)**
PANSS_P	-	-	30.47 ± 5.34	74	26.65 ± 6.87	65	-	2.37(0.129)
PANSS_N	-	-	36.20 ± 5.41	74	34.20 ± 7.29	65	-	0.62(0.433)
PANSS_G	-	-	72.68 ± 7.28	74	68.58 ± 13.79	65	-	**46.82(<0.001)**
FPG (mmol/l)	5.18 ± 0.70	58	4.95 ± 0.92	73	4.61 ± 0.93	46	2.57(0.112)	**2.08(0.043)**
Insulin (μIU/ml)	6.58 ± 3.17	58	12.72 ± 11.14	65	22.08 ± 16.06	39	**16.40(<0.001)**	**-5.46(<0.001)**
QUICKI	0.68 ± 0.07	58	0.61 ± 0.10	64	0.53 ± 0.08	39	**16.56(<0.001)**	**6.08(<0.001)**
HOMA-IR	1.53 ± 0.79	58	2.82 ± 2.55	64	4.50 ± 3.51	39	**13.84(<0.001)**	**-3.70(<0.001)**
HOMA-ISI	-3.44 ± 0.41	58	-3.88 ± 0.69	64	-4.40 ± 0.64	39	**18.13(<0.001)**	**5.92(<0.001)**
G/I	0.91 ± 0.30	58	0.58 ± 0.31	64	0.32 ± 0.25	44	**35.67(<0.001)**	**5.33(<0.001)**
TCHO (mmol/l)	4.57 ± 0.77	58	4.59 ± 1.09	71	4.45 ± 0.73	45	0.01(0.907)	0.73(0.469)
TG (mmol/l)	1.35 ± 0.88	58	1.56 ± 1.24	69	1.43 ± 0.65	45	1.17(0.282)	-0.20 (0.845)
HDL (mmol/l)	1.38 ± 0.36	58	1.29 ± 0.31	68	1.22 ± 0.29	45	2.71(0.102)	1.95(0.058)
LDL (mmol/l)	2.53 ± 0.71	58	2.83 ± 0.80	69	2.68 ± 0.69	45	**5.09(0.026)**	0.05(0.958)
LEP (ng/ml)	8.98 ± 5.62	58	10.88 ± 7.35	70	11.67 ± 1.09	47	2.59(0.110)	**-2.16(0.036)**
APN (μg/ml)	9.71 ± 0.96	58	9.39 ± 0.85	68	9.36 ± 0.82	49	**4.14(0.044)**	1.35(0.184)

**Note**: Data presented as mean ± standard deviation. Significant differences are highlighted in bold. ^a^The comparisons between FEAN-SCZ patients at baseline and healthy controls, after Bonferroni correction. ^b^The comparisons between FEAN-SCZ patients at baseline and follow up, using analysis of covariance. **Abbreviations**: APN, Adiponectin; BMI, Body Mass Index; FEAN, first-episode and antipsychotic-naïve; FPG, Fasting plasma glucose; G/I, FPG/Insulin; HDL, High-density lipoprotein; HOMA, Homeostasis model assessment; IR, Insulin resistance index; ISI, Insulin sensitivity index; LEP, Leptin; LDL, Low-density lipoprotein; PANSS, Positive and Negative Syndrome; PANSS_P, PANSS positive symptoms; PANSS_N, PANSS negative symptoms; PANSS_G, PANSS general psychopathological symptoms; QUICKI, Quantitative insulin sensitivity check index; SCZ, schizophrenia; TCHO, Total-Cholesterol; TG, Triglyceride.

## Data Availability

Not applicable.

## LIST OF ABBREVIATIONS

AAPD: Atypical Antipsychotic Drugs
CLO: Clozapine
FPG: Fasting Plasma Glucose
HDL: High-density Lipoprotein
IR: Insulin Resistance
LDL: Low-density Lipoprotein
OLA: Olanzapine
SCZ: Schizophrenia
